# An in vivo morphometry study on the standard transsylvian trajectory for mesial temporal lobe epilepsy surgery

**DOI:** 10.1186/s40064-015-1198-x

**Published:** 2015-08-09

**Authors:** Tadashi Hamasaki, Toshinori Hirai, Kazumichi Yamada, Jun-ichi Kuratsu

**Affiliations:** Department of Neurosurgery, Kumamoto University Medical School, 1-1-1 Honjo, Chuo-ku, Kumamoto, 860-8556 Japan; Department of Diagnostic Radiology, Kumamoto University Medical School, 1-1-1 Honjo, Chuo-ku, Kumamoto, 860-8556 Japan; Department of Radiology, Faculty of Medicine, University of Miyazaki, 5200 Kiyotake, Miyazaki, 889-1692 Japan

**Keywords:** Epilepsy surgery, Morphometry, Temporal lobe epilepsy, Transsylvian approach

## Abstract

A safe and appropriate surgical approach to the medial temporal structure is a prerequisite to perform surgeries for temporal lobe epilepsy. We used in vivo morphometry to identify the standard direction for entry into the inferior horn of the lateral ventricle via the Sylvian fissure: an important initial step in performing transsylvian selective amygdalohippocampectomy. 3D magnetic resonance images obtained from 28 patients without intra-parenchymal lesions were re-oriented to demonstrate all points in the Talairach space of the brain. The limen insulae and the midpoint between the hippocampal sulcus and the innominate sulcus on the coronal slice through the posterior edge of the amygdala were defined as the start and target points, respectively. We evaluated the direction of the vector between these two points and its validity in the brain of 12 patients with temporal lobe epilepsy. The direction of the mean approach vector was 52.4° posteriorly and 16.2° inferiorly. The mean approach vector on the axial plane showed the approximate parallelism with the sphenoid ridge in individual cases. The computer simulation revealed that our average approach vector correctly entered the inferior horn of the lateral ventricle in all temporal lobe epilepsy brains. In vivo morphometry may contribute to the further development of safe and minimally-invasive neurosurgical procedures.

**Electronic supplementary material:**

The online version of this article (doi:10.1186/s40064-015-1198-x) contains supplementary material, which is available to authorized users.

## Background

The medial temporal structure, i.e. the hippocampus, amygdala, and parahippocampal gyrus, is involved in several neurological disorders including temporal lobe epilepsy (TLE). Surgery for refractory TLE results in long-term seizure control in more than 70% of patients (Cohen-Gadol et al. 2006; Morino et al. 2009; Yasargil et al. 1993; Sindou et al. 2006; Engel 1996) and is superior to the best currently available medical treatments (Wiebe et al. 2001). While the optimal surgical approach to achieve better seizure outcomes is still matter of debate (Josephson et al. 2013; Hu et al. 2013), selective approaches to the medial temporal region attempt to spare the anterior temporal cortices and underlying white matter (Morino et al. 2009; Hori et al. 2007; Bujarski et al. 2013). Transsylvian selective amygdalohippocampectomy (TSA)(Wieser 1986; Yasargil et al. 1985) is unique in that it does not need to dissect the temporal lobe cortex while other selective approaches such as transcortical- (Olivier 2000; Niemeyer 1958) or subtemporal approach (Hori et al. 1993) need to. TSA is thought to be technically difficult and to require surgical expertise (Yonekawa 2007). The disadvantages include limited exposure of medial temporal structures, the risk of iatrogenic vascular injury or vasospasm of the middle cerebral arteries (Schaller et al. 2004), and the risk to injure the perimesencephalic vessels or even enter into the brain stem if the dissection went too medial in the depth. An important initial step in performing TSA is to take a precise direction of dissection through the most inferior portion of the insular cortex and underlying temporal stem for entry into the inferior horn. Intraoperative navigation, however, is not informative in most cases because considerable brain shift happens after drainage of the cerebrospinal fluid inside the Sylvian fissure (Morino 2013). The anatomical study showing the standard approach direction has been scarce.

Advances in the spatial resolution of clinical neuroimaging scanners such as 3T magnetic resonance (MR) imaging instruments and statistical techniques such as SPM (available at http://www.fil.ion.ucl.ac.uk/spm/) that can analyze subtle differences in 3D structures facilitate large-sample in vivo studies of the living human brain (Good et al. 2001). In vivo morphometry has been used to study statistical changes in brain structures in patients with central nervous system disorders such as Parkinson’s disease (Hamasaki et al. 2010), schizophrenia (Job et al. 2002), focal cortical dysplasia (Colliot et al. 2006), and temporal lobe epilepsy (Pail et al. 2012). The technique helps to investigate individual variations in important structures such as the location of the central sulcus (Hamasaki et al. 2012) in normal subjects, information useful for planning neurosurgical procedures.

**Fig. 1:**
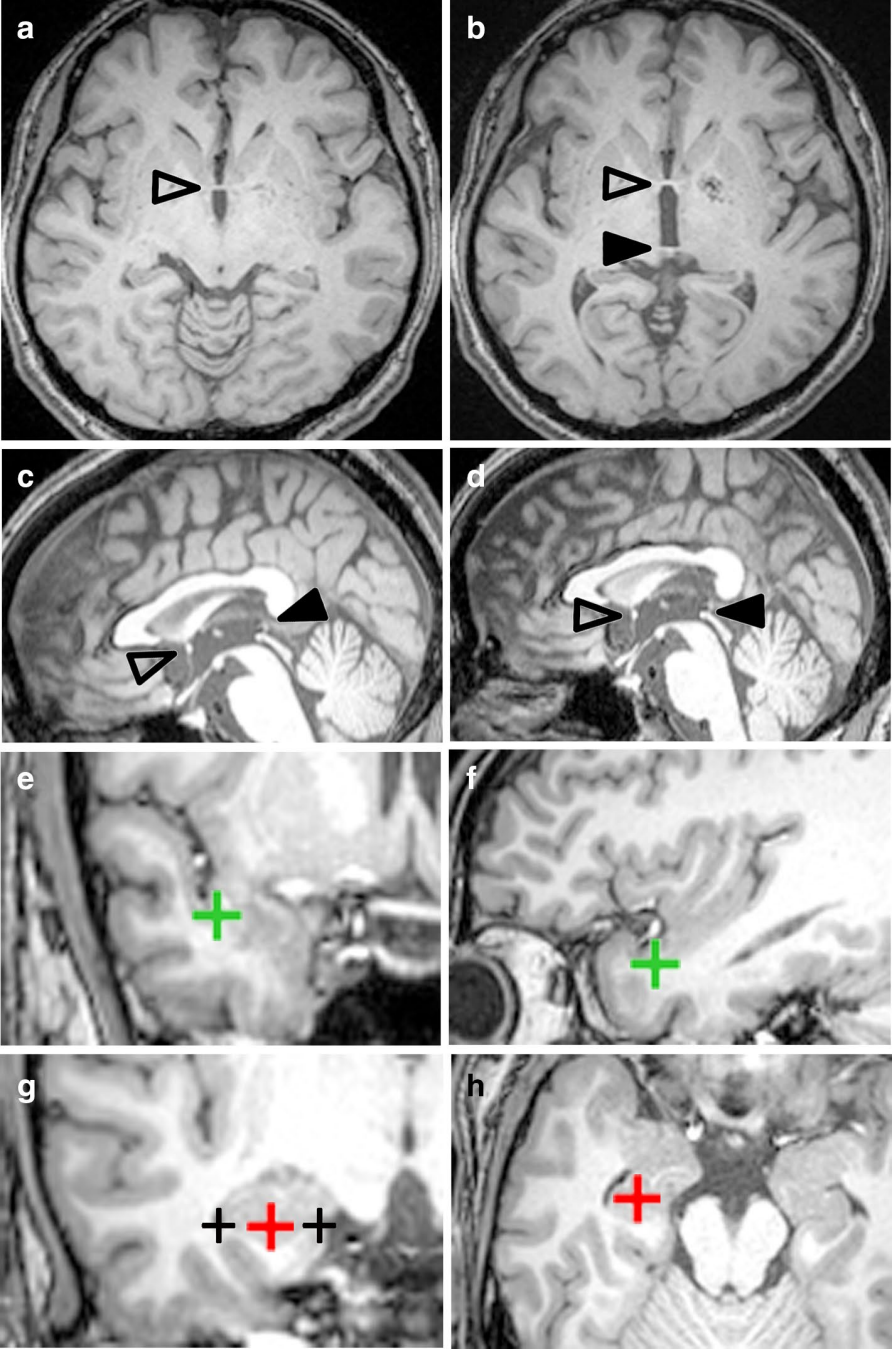
Procedures for image analysis. Raw data from MPRAGE sequences (**a**, **c**) were reoriented to place the *line* of the anterior-posterior commissure (AC *open arrowheads*; PC *arrowheads*) on both the horizontal (**b**) and the mid-sagittal (**d**) planes. The limen insulae was pointed to on the most anterior coronal slice on which the temporal stem was recognized (*green cross* in **e**). The accuracy of the point placement was confirmed on a sagittal slice (*green cross* in **f**). The target (*red cross* in **g**) was pointed to the midpoint between the hippocampal sulcus (*black cross* in the medial in **g**) and the innominate sulcus (*black cross* in the lateral in **g**) on the coronal slice through the posterior edge of the amygdala. Its location was also confirmed on an axial slice (*red cross* in **h**).

**Fig. 2:**
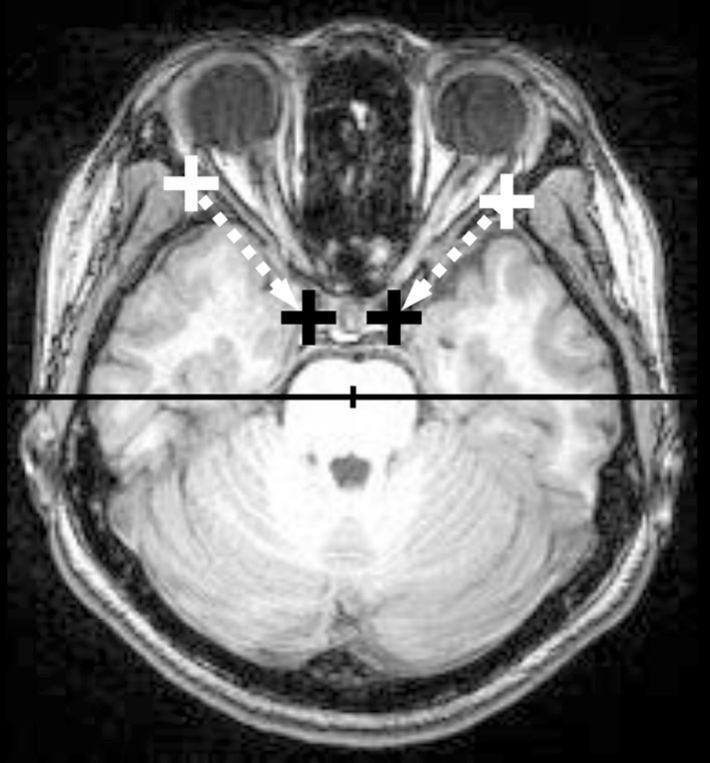
The direction of the sphenoid ridge on the axial plane. The *line* on the sphenoid ridge was drawn (*dashed lines*) between the outer point near the pterion (*white marks*) and the inner point close to the clinoid process (*black marks*). The angle between the sphenoid ridge line and x-axis of the coordinates (a *horizontal line*) was calculated as described in the "Methods” section.

To identify the standard transsylvian trajectory to enter the inferior horn of the lateral ventricle we performed in vivo morphometry analysis on brain imaging data (Fig. 1) acquired in subjects without intraparenchymal organic lesions. We tested the parallelism of the approach angle with the sphenoid ridge (Fig. 2), which is always identifiable in the operative field and a potential reference line in practice. We also evaluated whether our standard trajectory was valid in the brain of patients with TLE.

## Results

The male:female ratio in our study population for the trajectory analysis was 3:4. There were 15 patients with trigeminal neuralgia, 11 with hemifacial spasm, and one each with painful tic convulsif and vascular compression syndrome of the vestibulocochlear nerve. The mean age of our 28 patients was 60.8 ± 15.1 years (range 16–82 years). For the surgical simulation analysis on 12 TLE patients, there were 5 males and 7 females and the mean age was 31.9 ± 11.0 years (range 17–58 years). Seven patients had hippocampal sclerosis.

The mean location of the limen insulae (Fig. 1e, f) and the target point of the hippocampus (Fig. 1g, h) in the Talairach space is shown in Additional file 1: Table S1. There was no significant laterality in the x-coordinate of the limen insulae. The y- and the z-coordinate of the limen insulae was significantly smaller on the left than on the right. Our data indicate that the limen insulae was located more postero-inferiorly on the left than on the right, confirming previously-reported significant right-left differences in the Sylvian fissure (Thompson et al. 1998).

**Fig. 3:**
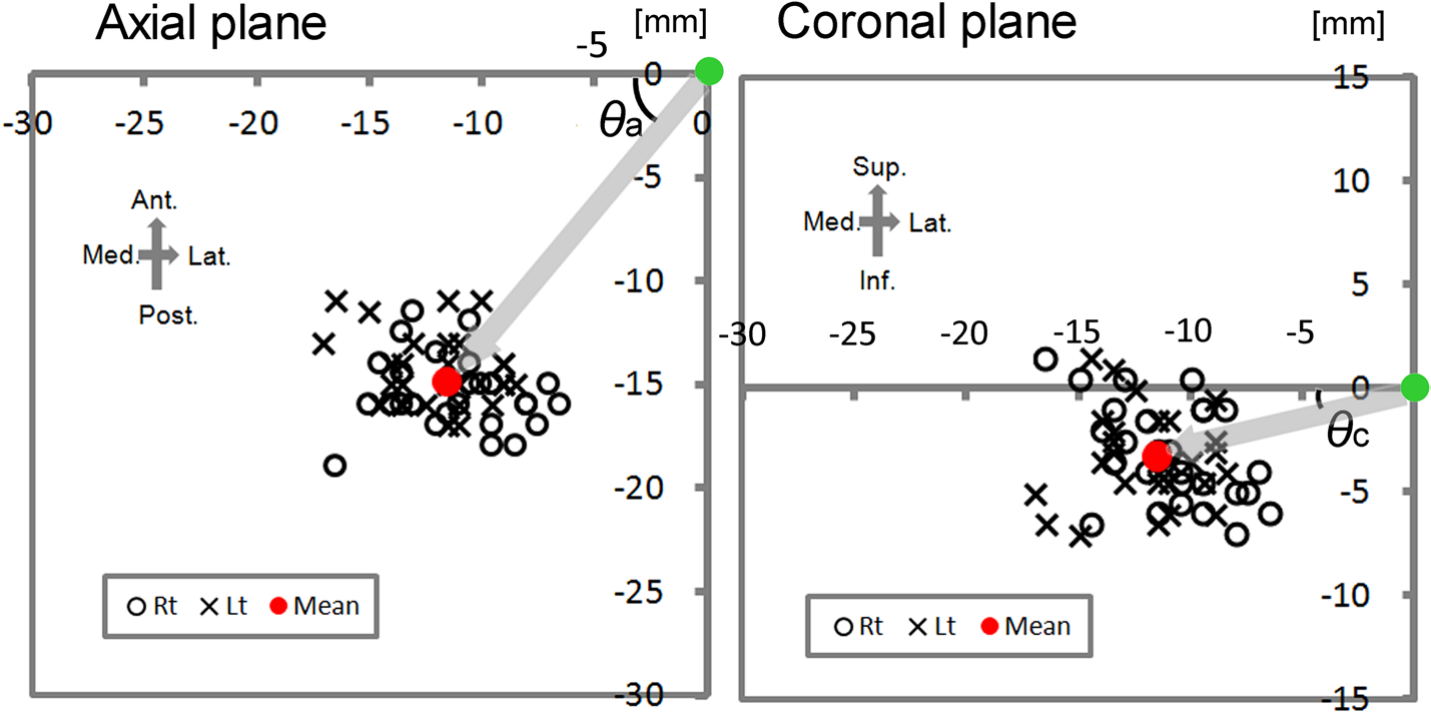
Scatter plots of the anatomical relationship between the limen insulae and the target. In the *left panel* the location of the target (*open circles* for the *right*-, x-marks for the *left*-, and *red dot* for the mean location) was plotted on the coordinate axis defined on the axial plane on which the limen insulae (a *green dot*) was set as the origin. The angle between the *right*-*left* axis (i.e., *x* axis) and the limen insulae-inferior horn vector (*gray arrow*) is defined as the approach angle on the axial plane (*θ*a). In the *right panel* the location of the target (*open circles* for the *right*-, x-marks for the *left*-, and *red dot* for the mean location) was plotted on the coordinate axis defined on the coronal plane on which the limen insulae (*green dot*) was set as the origin. The angle between the *right*-*left* axis (i.e., *x* axis) and the limen insulae-inferior horn vector (*gray arrow*) is defined as the approach angle on the coronal plane (*θ*c). The scale is shown in the *upper right corner* of each *panel*. The orientation is shown in the *upper left* corner. *Ant.* anterior, *Inf.* inferior, *Lat.* lateral, *Med.* medial, *Post.* posterior, *Sup.* superior.

**Fig. 4:**
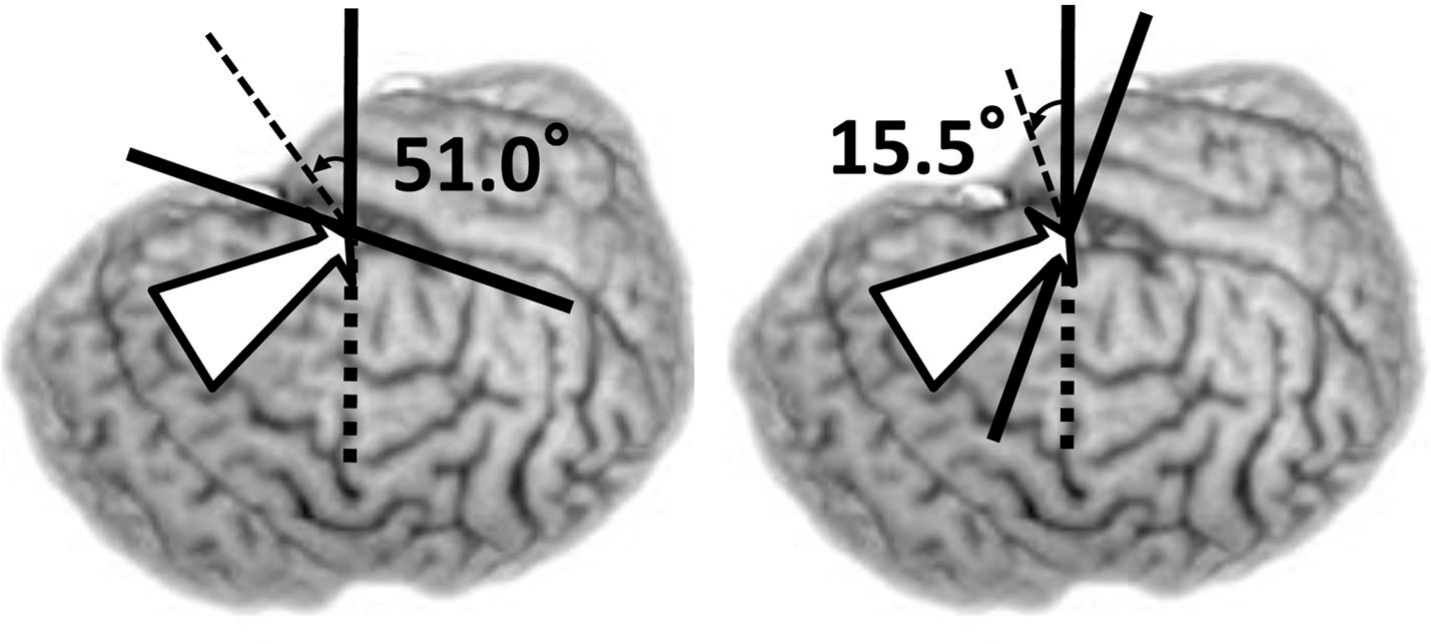
The standard approach vector superimposed on the 3D brain image of 35-year-old TLE patient. When the standard vector (*white arrow*) is expressed in the chiasmatico-commissural coordinate, where the horizontal plane is parallel to the sylvian fissure, it was oriented 51.0° posteriorly along the sylvian fissure (*left*) and 15.5° in inferiorly along the axis perpendicular to the Sylvian fissure (*right*).

On the axial plane the mean approach angle (Additional file 1: Table S2 and *θ*a in the left graph in Fig. 3) was 52.4°. The maximum and minimum angles were 67.9° and 33.7°; the range was 34.2°. The mean angle was significantly larger (p < 0.01, paired-*t* test) on the right- (54.6°, Additional file 1: Table S2 and open circles in Fig. 3) than on the left side (50.2°, Additional file 1: Table S2 and x-marks in Fig. 3). On the coronal plane, the mean approach angle (Additional file 1: Table S2 and *θ*c in the right graph in Fig. 3) was 16.2°. The maximum and minimum angles were 42.7° and −5.9°; the range was 48.6° (Additional file 1: Table S2). We found no statistically significant difference (p = 0.392, paired *t* test) between the mean angle on the right (17.0°, Additional file 1: Table S2 and open circles in Fig. 3) and left (15.5°, Additional file 1: Table S2 and x-marks in Fig. 3). When the average approach vector was transferred to the chiasmatico-posterior commissural (CH-PC) coordinate, it was oriented 51.0° posteriorly along the sylvian fissure and 15.5° in inferiorly along the axis perpendicular to the Sylvian fissure (Fig. 4).

**Fig. 5:**
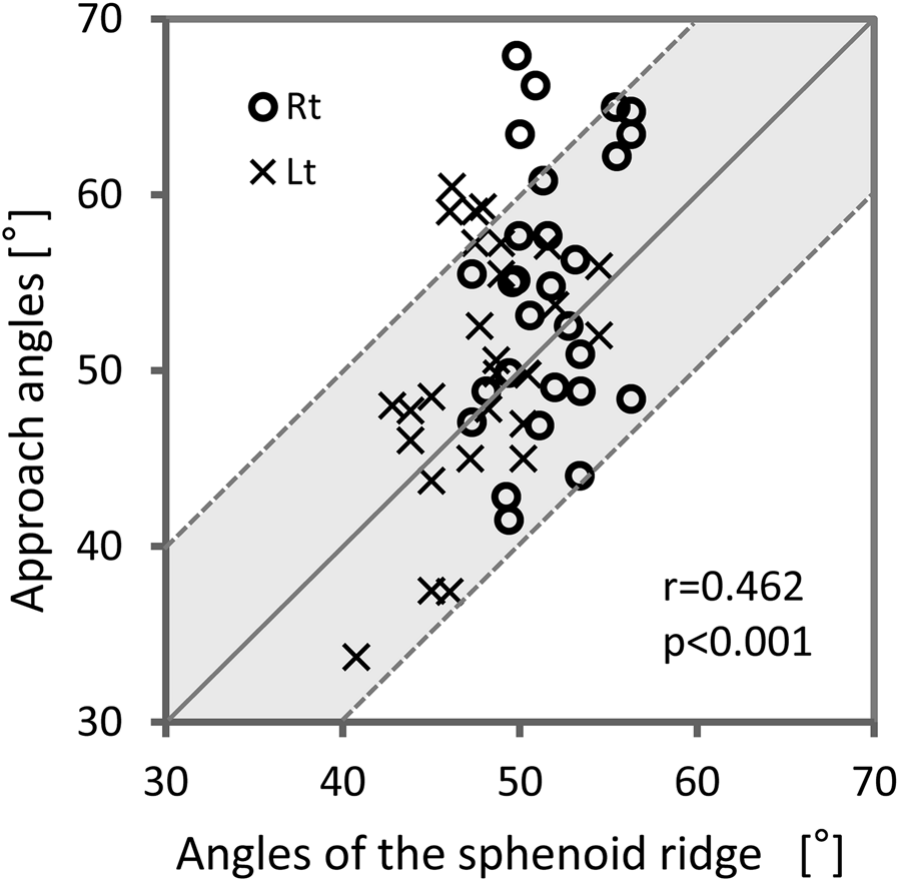
Scatter plot of the approach angle on the axial plane (y-axis) plotted against the angle of the sphenoid ridge (x-axis) in individual cases. *Open circles* and x-marks are for the *right* and *left sides*, respectively. The Pearson correlation coefficient (r) was significant at the 0.001 level. The *diagonal line* indicate the exact parallelism between the sphenoid ridge and the approach angle. Two *dotted lines* indicate that the difference between these two angles are ±10°. This plot illustrates the close parallelism between two angles within individuals: the most of the appropriate approach angle (89.3%; 50/56) were oriented within ±10° along the direction of the sphenoid ridge.

We found the large range of deviation from the mean approach angles (Fig. 3; Additional file 1: Table S2). Thus, we examined the parallelism between the standard approach vector and the sphenoid ridge to test whether the sphenoid ridge can be used as a reference line for approach direction. The mean angle between the sphenoid ridge and x axis of the Talairach coordinate on axial section (Fig. 2) was 49.7° ± 3.5° (range, 40.8° to −56.3°). We found significant correlation between the approach angle and the angle of the sphenoid ridge within individuals (Fig. 5; r = 0.462, p < 0.001; univariate analysis, Pearson linear correlation). Furthermore, the most of the appropriate approach angle (89.3%; 50/56) were oriented within ±10° along the direction of the sphenoid ridge (Fig. 5). Our data clearly demonstrated that there are close parallelism of the approach vector with the sphenoid ridge.

**Fig. 6:**
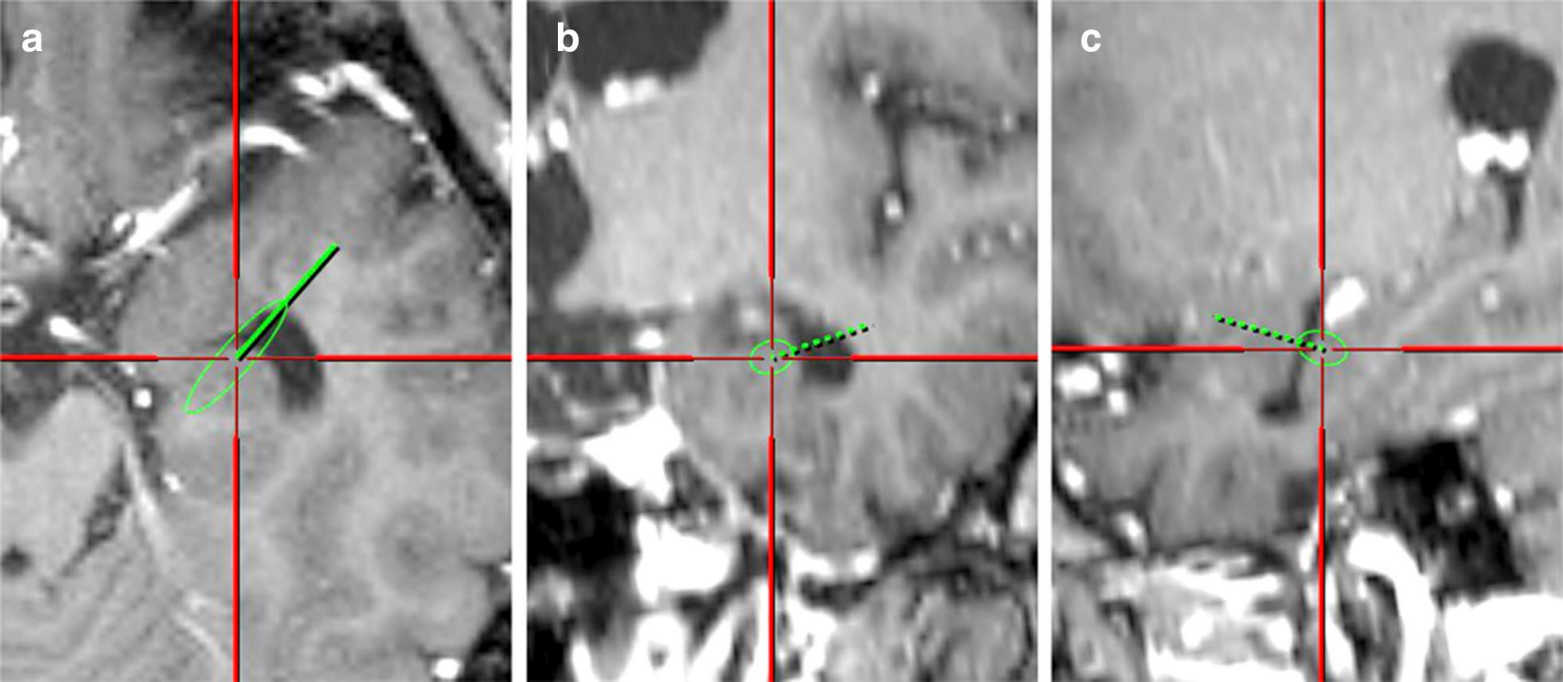
Images of surgical simulation demonstrating the validity of our standard approach vector. We analyzed 3D MR images of our TLE patients using computer simulation and found that the vector successfully entered the inferior horn in the axial (**a**), coronal (**b**), and sagittal (**c**) slices.

Finally, the surgical simulation analysis showed that our average approach angle (i.e., 52.4° posteriorly and 16.2° inferiorly in the Talairach coordinate) from the limen insulae successfully entered the inferior horn of the lateral ventricle in all of our 12 patients with TLE (Fig. 6).

## Discussion

We performed in vivo morphometry analysis on 3D high-resolution structural brain images to investigate the transsylvian surgical route to the human medial temporal structure. When the limen insulae was set as the entry point, the direction of the mean approach vector was 52.4° posteriorly and 16.2° inferiorly (Fig. 3). There was close parallelism of the approach vector with the direction of the sphenoid ridge: the difference of these two mean angles on the axial plane was as less as 2.7°. We found a linear correlation between these two angles at the individual level (Fig. 5). Our simulation analysis demonstrated that our vector was valid and can be useful to estimate the appropriate trajectory during surgery for TLE patients (Fig. 6).

An important early step in the transsylvian transsylvian surgical approach to the medial temporal structure is the dissection of the white matter underlying the inferior limiting sulcus of the insula known as the temporal stem. Since severe dysfunction of the temporal stem results in various cognitive deficits and visual field defects (Wang et al. 2008), a straightforward and minimally invasive approach is desirable especially on the language-dominant side (Morino et al. 2009; Wang et al. 2008). Compared to vascular surgeries such as aneurysm clipping, the number of surgical cases of TSA that one neurosurgeon can experience is limited. It should be a stressful procedure, especially for surgeons who have not performed a number of TSA surgeries, to dissect the temporal stem by visual estimation, so that it would be useful to show the standard approach vector. Our analysis indicated that the dissection parallel with the direction of the sphenoid ridge would enter the inferior horn within 10° errors in most cases. Together with our results, the image analysis by patient-specific virtual models and advanced 3D reconstruction techniques for clinical imaging (Malone et al. 2010; Robison et al. 2011; Stadie et al. 2008) can help to address this issue.

A problem we encounter in using intraoperative neuronavigation is often caused by the brain shift after CSF drainage. If the entry point, i.e., the limen insulae, has been already shifted considerably after opening the Sylvian fissure, the vector from the shifted entry point to the target point, which is estimated by navigation, can be far from the correct trajectory. The standard vector is still valid as long as the target point is shifted in the same way as the entry point, suggesting that our approach would warrant higher accuracy than neuronavigation. Our statistical analysis also showed that the direction of the sphenoid ridge showed close parallelism with the correct trajectory within individuals (Fig. 5) although there were variations in the trajectory across individuals (Fig. 3). The next study is underway to address whether these findings are useful in surgical practice just as shown by the simulation (Fig. 6).

The asymmetry of human brain structures has been reported (Good et al. 2001; Thompson et al. 1998; Geschwind and Galaburda 1985; Leroy et al. 2015; Witelson and Kigar 1992). Leroy et al. (2015) investigated 177 MR images of human brains and found that the superior temporal sulcus was deeper on the right than the left irrespective of handedness, language dominance, and sex. Witelson and Kigar (1992) studied the cortical structures of postmortem brains and found that the horizontal segment of the Sylvian fissure was greater on the left than on the right and that the vertical segment greater on the right. Thompson et al. (1998) who performed 3D statistical analysis on MR images from 10 normal aging subjects found that the horizontal extent of the Sylvian fissure was 8.8 mm longer on the left than on the right. They also documented that the vertical extent of the Sylvian fissure was 7.6 mm shorter on the left than on the right. The shape of the Sylvian fissure appeared to be elongated in the antero-posterior dimension on the left compared to the right. Our results showed the presence of asymmetry in the standard transsylvian trajectory: the mean angle on the axial plane on the right was directed more posteriorly than the left (Fig. 3; Additional file 1: Table S2). However, right-left differences may not need to be considered in the approach because the difference in the approach angle was negligibly small (4.4° on the axial plane, see Additional file 1: Table S2).

The intraoperative acquisition of anatomic information can be difficult due to time restrictions, the small size of the surgical field in some operations, and the stress experienced by senior and assisting neurosurgeons (Frati et al. 2006). Therefore, cadaveric dissection plays a key role not only for surgical training but also for the refinement of microsurgical techniques and the development of new surgical approaches and instruments. We suggest that in vivo morphometry offers certain advantages. While the 3D structures of cadaveric brains can be affected by fixation (Messert et al. 1972), in vivo morphometry yields absolute measurements in living brains, group studies on large patient populations are possible, and the measurements can be repeated as needed to obtain accurate results. Visual inspection of a large number of brains with structural variability is needed for the neurosurgeon to construct 3D brain image concepts ranging from macroscopic- to microsurgical levels.

## Conclusions

We performed in vivo morphometry analysis and the acquired 3D anatomical information on the transsylvian surgical trajectory to the medial temporal lobe. We propose the standard transsylvian trajectory (Figs. 3, 4), which showed close parallelism with the sphenoid ridge on the axial plane and may be useful in surgical practice. We suggest that the information generated from in vivo morphometry yields further insight into the human neurosurgical anatomy and may contribute to the development of safe and minimally-invasive neurosurgical procedures.

## Methods

### Enrollment and acquisition of 3D MR images

We declare that all human studies have been approved by our Ethics Committee and have therefore been conducted in accordance with the Declaration of Helsinki in 1964 and its later amendments. We declare that all patients gave informed consent prior to inclusion in this study. We enrolled 28 consecutive patients with neurovascular compression syndrome diagnosed between August 2011 and October 2013 because none of them had intra-parenchymal organic lesions. We expected some of our patients to manifest age-related brain atrophy, which would approximate the brains of patients with epilepsy (Bonilha et al. 2006; Bernasconi et al. 2004). There were some reports on cadaveric brains that showed the difference of Sylvian fissure morphology in relation to handedness (Witelson and Kigar 1992). However, Good et al. (2001) studied a large number of MR images by voxel-based morphometry and found no significant difference in the bilateral hemispheres reflective of handedness, so that we did not separate left-, mixed-, and right-handed subjects. We performed brain scanning on a 3T clinical MR imager (Magnetom Trio; Siemens AG, Erlangen, Germany) using an 8-channel phased-array head coil. Magnetization-prepared rapid gradient-echo (MPRAGE) sequences yielded T1-weighted volume data. The parameters for MPRAGE imaging were repetition time, 1,900 ms; effective echo time, 4.7 ms; inversion time, 900 ms; imaging time, 4 min 18 s. All images were acquired with a 23 × 23-cm field of view, a 256 × 256 matrix, and one excitation. MPRAGE images obtained from 12 patients with TLE during the same period were also enrolled to evaluate the standard approach vector.

### Pre-processing and morphometry of imaging data

Raw MR imaging data were pre-processed for in vivo morphometry study according to the reports published previously (Hamasaki et al. 2010, 2012; Filipek et al. 1989). The anterior-posterior commissural (AC-PC) line was on both the mid-sagittal plane and the plane of the axial slices (Fig. 1a–d). This process showed all points placed on the brain image in the Talairach coordinate system (Talairach and Tournoux 1988). The limen insulae, the starting point of the insular cortex in the basal part of the Sylvian fissure, is normally close to the bifurcation of the middle cerebral artery and the site of corticotomy for standard TSA (Yaşargil et al. 1985). The MRIcro software (available at http://www.cabiatl.com/mricro/mricro/index.html) calculated the coordinate of the axis of the limen insulae location on the pre-processed 3D MR images that contained the point placed on the temporal stem on the most anterior coronal slice on which the temporal stem was first identifiable (green crosses in Fig. 1e, f). We defined the midpoint between the hippocampal sulcus and the innominate sulcus on the coronal slice through the posterior edge of the amygdala (red crosses in Fig. 1g, h) as the target point because it is in the area where the inferior horn is almost always identifiable (Fig. 1g, h). The location of the limen insulae and the target point was then expressed in terms of the Talairach coordinate system where the location of anterior commissure is set as the origin. The sphenoid ridge was defined as the reference line to estimate the approach trajectory in the operative field. The line was drawn (dashed lines in Fig. 2) between the outer point near the pterion (white marks in Fig. 2) and the inner point close to the clinoid process (black marks in Fig. 2) on the axial slice through the sphenoid ridge.

To analyze the average approach vector we constructed another 3D space where the limen insulae was set as the origin because it is the starting point of the vector (Fig. 3). The location of the target point was then plotted on the coordinate axis defined separately on the axial and coronal planes so that the approach vector from the limen insulae to the target point was independently expressed in these two planes (see Fig. 3). The approach angle on the axial plane (*θ*a in Fig. 3) was defined as the angle between the x axis and the approach vector radiating on the axial plane, and calculated by the standard trigonometry (Ishihara 1993). The approach angle on the coronal plane (*θ*c in Fig. 3) was defined as the angle between the x axis and the approach vector radiating on the coronal plane. Finally, we transferred the mean approach vector to the CH-PC coordinate, which is defined by the horizontal reference line through the superior border of the chiasm and the inferior border of the PC (Tamraz and Comair 2000). The CH-PC line is 18.2° oblique to the AC-PC line and can be identified in the operative field because the line is oriented parallel to the sylvian fissure (Tamraz and Comair 2000). The approach angle in the CH-PC coordinate were calculated by trigonometry (Ishihara 1993). The direction of the sphenoid ridge was defined by the angle between the line of the sphenoid ridge and x axis of the coordinates (a horizontal line in Fig. 2).

### Computer simulation of the standard surgical trajectory in TLE patients

To evaluate whether the standard trajectory is valid in the patients with TLE, we used FrameLink system^®^ of StealthStation^®^ (Medtronics). We inputted the location of the entry point (i.e., limen insulae) and the standard approach angles into the workstation and tested whether the trajectory entered into the inferior horn of the lateral ventricle.

### Statistical analysis

To evaluate the right-left difference we used the paired *t*-test. The correlation between the approach angle and the angle of the sphenoid ridge on the axial plane was assessed by univariate analysis (Spearman’s nonparametric rank correlation). All statistical analyses were with SPSS 10J^®^ software (SPSS, Chicago, IL, USA) running on a PC. A p value of <0.01 was considered significant.

## Additional file

Additional file 1:
**Table S1.** Location of the limen insulae and the target point at the hippocampus in the Talairach coordinate system. **Table S2.** Approach angle of the transsylvian trajectory to the medial temporal structure.

## Abbreviations

AC-PC: anterior-posterior commissural
CH-PC: chiasmatico-commissural
MR: magnetic resonance
TLE: temporal lobe epilepsy
TSA: transsylvian selective amygdalohippocampectomy
