# Supplementary material for: An in vivo morphometry study on the standard transsylvian trajectory for mesial temporal lobe epilepsy surgery
Source: Springerplus. 2015 Aug 9;4:406. doi: 10.1186/s40064-015-1198-x (PMC4529845; doi:10.1186/s40064-015-1198-x)
Supplement: Additional file 1: — Table S1. Location of the limen insulae and the target point at the hippocampus in the Talairach coordinate system. Table S2. Approach angle of the transsylvian trajectory to the medial temporal structure. [file 40064_2015_1198_MOESM1_ESM.docx]

**Table S1** Location of the limen insulae and the target point at the hippocampus in the Talairach coordinate system

|  | Limen insulae | | | Target point at the hippocampus | | |
| --- | --- | --- | --- | --- | --- | --- |
|  | x | y | z | x | y | z |
| Rt | 36.6±2.1 | 6.2±2.1 | -14.3±2.4 | 25.5±1.8 | -9.3±1.9 | -17.4±1.7 |
| Lt | -35.9±2.1 | 3.0±2.0** | -15.5±2.0** | -23.9±2.2** | -11.3±1.8** | -18.8±1.6** |

Unit, mm. **p < 0.01, paired *t*-test for right-left differences.

**Table S2** Approach angle of the transsylvian trajectory to the medial temporal structure

|  | Axial plane ^a^ | | | Coronal plane ^b^ | | |
| --- | --- | --- | --- | --- | --- | --- |
|  | Right | Left | Total | Right | Left | Total |
| Mean [°] | 54.6 | 50.2** | 52.4 | 17.0 | 15.5 | 16.2 |
| S.D. [°] | 7.5 | 7.0 | 7.5 | 13.5 | 10.0 | 11.8 |
| Maximum [°] | 67.9 | 60.5 | 67.9 | 42.7 | 33.7 | 42.7 |
| Minimum [°] | 41.5 | 33.7 | 33.7 | -5.2 | -5.9 | -5.9 |
| Range (max - min) [°] | 26.4 | 26.8 | 34.2 | 47.9 | 39.6 | 48.6 |

^a^ Positive values indicate that the angle is directed to the posterior from the right-left axis when the limen insulae and the midpoint between the hippocampal sulcus and the innominate sulcus on the coronal slice through the posterior edge of the amygdala are set as the entry and target points, respectively. ^b^ Positive values indicate that the angle is directed to the inferior. **p < 0.01, paired *t*-test for right-left differences. Abbreviations: S.D., standard deviation; max., maximum; min., minimum.
